# The Influence of Prenatal Fumonisin Exposure on Bone Properties, as well as OPG and RANKL Expression and Immunolocalization, in Newborn Offspring Is Sex and Dose Dependent

**DOI:** 10.3390/ijms222413234

**Published:** 2021-12-08

**Authors:** Ewa Tomaszewska, Halyna Rudyk, Izabela Świetlicka, Monika Hułas-Stasiak, Janine Donaldson, Marta Arczewska, Siemowit Muszyński, Piotr Dobrowolski, Iwona Puzio, Volodymyr Kushnir, Oksana Brezvyn, Viktor Muzyka, Ihor Kotsyumbas

**Affiliations:** 1 Department of Animal Physiology, Faculty of Veterinary Medicine, University of Life Sciences in Lublin, Akademicka St. 12, 20-950 Lublin, Poland; iwona.puzio@up.lublin.pl; 2 State Scientific Research Control Institute of Veterinary Medicinal Products and Feed Additives, Donetska St. 11, 79000 Lviv, Ukraine; galusik.77@gmail.com (H.R.); wolodjak@gmail.com (V.K.); brezvun@gmail.com (O.B.); muzyka@scivp.lviv.ua (V.M.); dir@scivp.lviv.ua (I.K.); 3 Department of Biophysics, Faculty of Environmental Biology, University of Life Sciences in Lublin, Akademicka St. 13, 20-950 Lublin, Poland; marta.arczewska@up.lublin.pl (M.A.); siemowit.muszynski@up.lublin.pl (S.M.); 4 Department of Functional Anatomy and Cytobiology, Faculty of Biology and Biotechnology, Maria Curie-Sklodowska University, 19 Akademicka St., 20-033 Lublin, Poland; monhul@o2.pl (M.H.-S.); piotr.dobrowolski@umcs.lublin.pl (P.D.); 5 Faculty of Health Sciences, School of Physiology, University of the Witwatersrand, 7 York Road, Parktown, Johannesburg 2193, South Africa; janine.donaldson@wits.ac.za

**Keywords:** fumonisins, newborns, rats, bone and cartilage, osteoprotegerin, receptor activator of nuclear factor kappa-Β ligand, expression

## Abstract

The current study examined the effects of exposure of pregnant dams to fumonisins (FBs; FB1 and FB2), from the seventh day of pregnancy to parturition, on offspring bone metabolism and properties. The rats were randomly divided into three groups intoxicated with FBs at either 0, 60, or 90 mg/kg b.w. Body weight and bone length were affected by fumonisin exposure, irrespective of sex or dose, while the negative and harmful effects of maternal FBs’ exposure on bone mechanical resistance were sex and dose dependent. The immunolocalization of osteoprotegerin (OPG) and receptor activator of nuclear factor kappa-Β ligand (RANKL), in bone and articular cartilage, indicated that the observed bone effects resulted from the FB-induced alterations in bone metabolism, which were confirmed by the changes observed in the Western blot expression of OPG and RANKL. It was concluded that the negative effects of prenatal FB exposure on the general growth and morphometry of the offspring bones, as a result of the altered expression of proteins responsible for bone metabolism, were dose and sex dependent.

## 1. Introduction

Besides the delivery of vitally important nutrients, the diet is also a source of other components, sometimes toxic ones, which make their way into the diet especially under favorable climatic conditions. Under such conditions, the feed can be contaminated with fungi like *Fusarium*, which produces mycotoxins as secondary products of its metabolism. These mycotoxins are called fumonisins (FBs). There are different types of FBs, including types A, B, C, and P, all of which are heat resistant. Fumonisins types B1 and 2 (FB1 and FB2) occur frequently and are of similar toxicity. FB1 and FB2 are found in over 50% of feed and feed raw material samples in a ratio of about 3:1, for FB1:FB2 [1,2,3]. *Aspergillus niger* has also been shown to produce FB metabolites and, for this reason, the consumption of FBs can be higher than expected [4]. The contamination of feed/nutrition with FB1 and FB2 is regulated by EU legislation [5,6]. Clinical signs observed in intoxicated subjects are dependent on FB dose, animal species, and the route of FB administration, as well as the sex and age of the animals [7]. Horses, pigs, sheep, and rodents are more sensitive to FBs than other animals and display non-species-specific symptoms like hepatic or kidney toxicity [8,9,10,11]. In horses and swine, certain organ-specific symptoms have been observed in target organs (like the brain in horses and the lungs or esophagus in swine) [12]. FBs have been shown to be hepatocarcinogenic in rats and mice, and they have also been shown to disturb the intestinal barrier even at a very low dose of 1.0 mg/kg b.w. [13,14].

FBs are considered potentially hazardous to humans, causing immunosuppression and neurotoxicity [15]. They are associated with stunting of growth in children and have been linked with folic acid deficiency-linked birth defects as a result of their interference with the uptake of folic acid [16,17,18]. The International Agency for Research on Cancer (IARC) has designated FB1 in Group 2B, meaning that FB1 is considered as “possibly carcinogenic to humans” [19]. The European Commission and the FDA in the USA have issued guidelines on the total amount of fumonisins acceptable in human foods and animal feed [6,20]. More than 70% of nutritional samples are contaminated with FBs in South America, Africa, and Southern Europe [21]. FBs have been found in commodities such as maize, dried figs, grapes, raisins, wine, coffee, and some plants [4,22]. Worldwide, human exposure to fumonisin B1 through maize is estimated to range between 0.01 to 354.0 μg/kg body weight [23].

Animal food and foods intended for infants, young children, or pregnant women can also be contaminated with FBs. Humans, unbeknownst to them, are commonly exposed to toxic compounds through their intake of food. Nutrition is known to affect prenatal development, with consequences often only evident later in life [24,25]. Taking into account that FBs cause various disturbances within an intoxicated living organism, it seems reasonable to investigate the effects of FB exposure during pregnancy on the bone metabolism of the fetus, which determines the development of the organism as a whole.

Therefore, the objective of this study was to determine the effects of maternal FB exposure on mechanical and geometric bone properties, as well as the expression and immunolocalization of osteoprotegerin (OPG) and receptor activator of nuclear factor kappa-Β ligand (RANKL), in the bone tissue and hyaline cartilage of both male and female newborn rats. In doing so, the present study examined (1) the general expression of the abovementioned proteins in bone tissue, (2) the changes in expression and immunolocalization of these proteins, as influenced by maternal FB exposure, (3) the detection of OPG and RANKL via Western blot, and (4) the thin collagen content in the bone trabeculae. Using standard immunohistochemistry methods, the immunoexpression of the selected proteins was established in articular cartilage, growth plate cartilage, trabecular, and compact bone. Together, these measurements should provide fundamental information regarding the outcomes of FB exposure during pregnancy on the bone metabolism of the offspring.

## 2. Results

**Figure 1:**
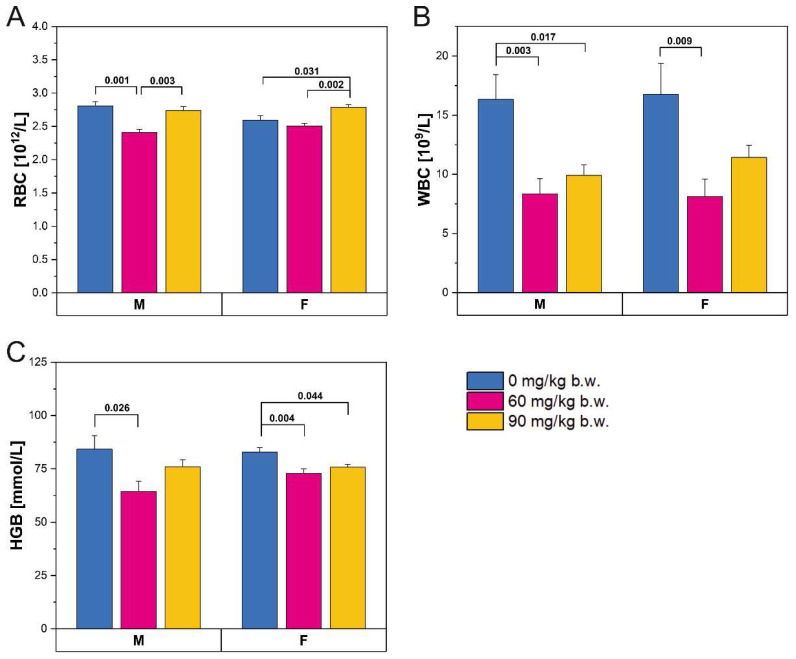
Red blood cell (RBC) count (**A**), white blood cell (WBC) count (**B**), and hemoglobin (HGB) concentration (**C**) in male (M) and female (F) newborn rats prenatally exposed to fumonisins. Data are given as mean ± standard error. M, male; F, female.

The administration of FBs affected the blood counts of both male and female rats (Figure 1). The number of red blood cells (Figure 1A) decreased slightly in male rats following consumption of the lower FBs’ dose and then returned to previous levels following consumption of the 90 mg/kg b.w. FB dose. A similar pattern was observed in the hemoglobin levels of the male rats (Figure 1C). The red blood cell count was increased in the female rats that received the high FBs’ dose, compared to those in the control group and those that received the low FBs’ dose (Figure 1A). Hemoglobin levels were significantly decreased in both FBs’ groups compared to control rats, irrespective of dose (Figure 1C). White blood cell count was decreased following FBs intoxication in both male and female rats, regardless of FBs’ dose (Figure 1B).

### 2.1. Body Weight and Mechanical and Geometric Bone Properties

Following FBs intoxication, a significant decrease in body weight, femur length, cross-sectional area, and ultimate force was observed in both sexes (Table 1). The changes observed were not dose dependent, except for the cross-sectional area. Femoral stiffness was significantly decreased in male rats following FBs intoxication, regardless of FBs’ dose, while no significant differences were observed between groups in the female rats. Interestingly, immature collagen content in trabecular bone was significantly decreased in male rats intoxicated with the higher FBs’ dose, while a significant increase in trabecular bone immature collagen content was observed in female rats, following consumption of the 90 mg/kg b.w. FBs’ dose. In compact bone, immature collagen content decreased in males following consumption of the 60 mg/kg b.w. FBs’ dose, while the 90 mg/kg b.w. FBs’ dose caused an increase in immature collagen content to a value similar to that observed in the control group. A dose-independent increase in immature collagen content in compact bone was observed in the female rats.

### 2.2. Immunostaining

**Figure 2:**
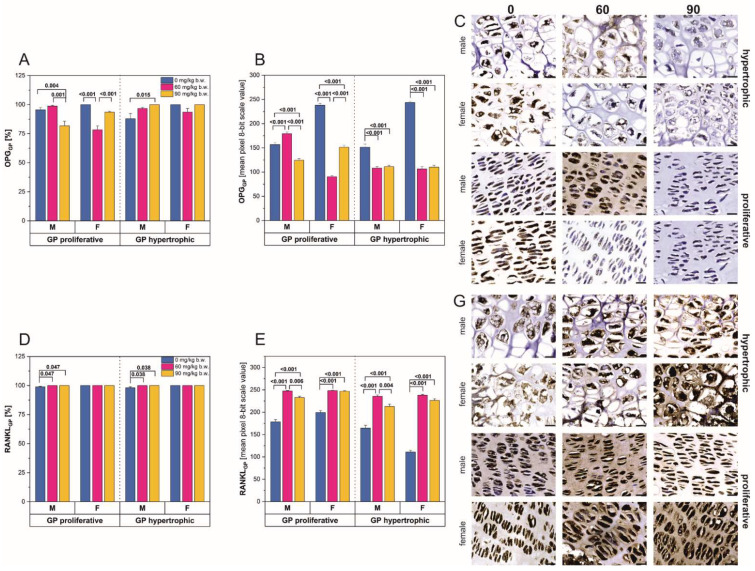
Analysis of the immunohistochemical reactions for osteoprotegerin (OPG) and receptor activator of nuclear factor kappa-Β ligand (RANKL) in the proliferative and hypertrophic zones of the femoral growth plate of male and female newborn rats prenatally exposed to fumonisins; (**A**,**D**) the percentage of cells with a positive response, (**B**,**E**) the intensity of the immunostaining reaction, measured by the quantitative assessment of the mean pixel intensity value in the photomicrographs converted to 8-bit, grayscale images, (**C**) representative pictures of the immunohistochemical analysis of OPG carried out on formaldehyde-fixed sections from the femoral growth plate cartilage of male and female newborn rats; (**G**) representative pictures of the immunohistochemical analysis of RANKL carried out on formaldehyde-fixed sections from the femoral growth plate cartilage of male and female newborn rats. Scale bars are equal to 20 µm. Data are presented as mean ± standard error. M, male; F, female; GP, growth plate.

The percentage of cells with positive immunostaining reactions for OPG (Figure 2A) and RANKL (Figure 2D) differed depending on the FBs’ dose. In male rats, the percentage of OPG-positive cells decreased significantly following intoxication with the higher FBs’ dose, within the proliferative region of the growth plate (Figure 2A). In contrast, the same FBs’ dose caused a statistically significant increase in the percentage of OPG-positive cells in the hypertrophic area, compared to that observed in the control rats. In both the proliferative and hypertrophic growth plate regions, a significant increase in percentage of RANKL-positive cells was observed in the male rats (Figure 2D). The lower FBs’ dose caused a significant decrease in the percentage of OPG-positive cells within the proliferative growth plate region of the female rats, while the 90 mg/kg b.w. FBs’ dose resulted in a percentage of OPG-positive cells similar to that observed in the control rats. No changes in the percentage of OPG-positive cells were observed in the hypertrophic region of the growth plate in female rats (Figure 2A). There were also no changes in the percentage of RANKL-positive cells between groups in both growth plate regions in the female rats.

FBs intoxication had a significant effect on the intensity of positive reactions for OPG (Figure 2B,C) and RANKL (Figure 2E,G). In the proliferative region of the growth plate (Figure 2B), male rats showed an initial increase in OPG staining reaction intensity following consumption of the 60 mg/kg b.w. FBs’ dose and a significant decrease following the 90 mg/kg b.w. FBs’ dose. In the hypertrophic region of the growth plate, a significant decrease in OPG reaction intensity was observed in male rats, regardless of FBs’ dose. The intensity of the OPG staining reaction observed in the female rats (Figure 2B,C) showed a different pattern compared to that of the males. OPG reaction intensity in the proliferative region of the growth plate was significantly reduced following the 60 mg/kg b.w. FBs’ dose, while the 90 mg/kg b.w. FBs’ dose significantly increased the intensity of the positive OPG reaction. In the hypertrophic region of the growth plate, OPG reaction intensity decreased significantly following FBs consumption, regardless of dose. The relative intensity of the RANKL-positive reactions (Figure 2E,G) was similar for both sexes and both growth plate regions and was increased compared to control rats, following both FBs’ doses. The reaction intensity was significantly reduced following consumption of the 90 mg/kg b.w. FBs’ dose compared to that observed in the rats following the 60 mg/kg b.w. FBs’ dose.

**Figure 3:**
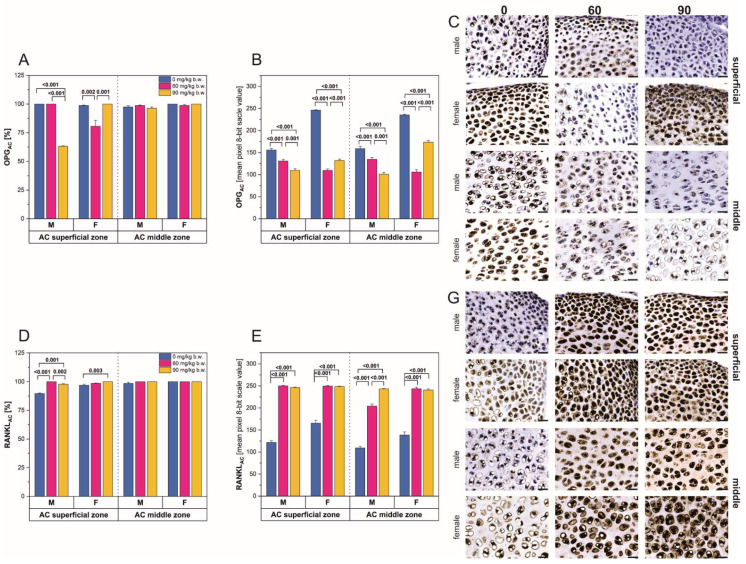
Analysis of the immunohistochemical reaction for osteoprotegerin (OPG) and receptor activator of nuclear factor kappa-Β ligand (RANKL) in the superficial and middle zones of the femoral articular cartilage of male and female newborn rats prenatally exposed to fumonisins; (**A**,**D**) the percentage of cells with a positive response, (**B**,**E**) the intensity of the immunostaining reaction, measured by the quantitative assessment of the mean pixel intensity value in the photomicrographs converted to 8-bit. grayscale images, (**C**) representative pictures of the immunohistochemical analysis of OPG carried out on formaldehyde-fixed sections from the femoral articular cartilage of male and female newborn rats; (**G**) representative pictures of the immunohistochemical analysis of RANKL carried out on formaldehyde-fixed sections from the articular cartilage of male and female newborn rats. Scale bars are equal to 20 µm. Data are presented as mean ± standard error. M, male; F, female; AC, articular cartilage.

In the superficial zone of the femoral articular cartilage (AC), prenatal intoxication with 90 mg/kg b.w. FBs caused a significant decrease in the number of OPG-positive cells in the male rats (Figure 3A). In contrast, the female rats showed a significant decrease in OPG-positive cells following consumption of the 60 mg/kg b.w. FBs’ dose and a significant increase following the 90 mg/kg b.w. FBs’ dose. For both sexes, no considerable changes in the number of OPG- or RANKL-positive cells were observed in the middle zone of the articular cartilage (Figure 3A,D), while in the superficial zone, a significant increase in the number of RANKL-positive cells was observed.

A significant decrease in the immunostaining reaction intensity for OPG was observed in all regions of the articular cartilage, in both male and female rats (Figure 3B,C). In the male rats, OPG staining intensity decreased with increasing level of FBs intoxication. A slightly different pattern was observed in the females, where the 60 mg/kg b.w. FBs’ dose decreased OPG staining intensity, while the higher FBs’ dose caused a slight increase in staining intensity compared to that observed in the female rats receiving the lower FBs’ dose. The immunostaining reaction intensity for RANKL was increased in both regions of the articular cartilage, in both male and female rats (Figure 3E,G). The increase in reaction intensity was not dose dependent in the superficial zone and middle zone of the articular cartilage in females (Figure 2 and Figure 3).

**Figure 4:**
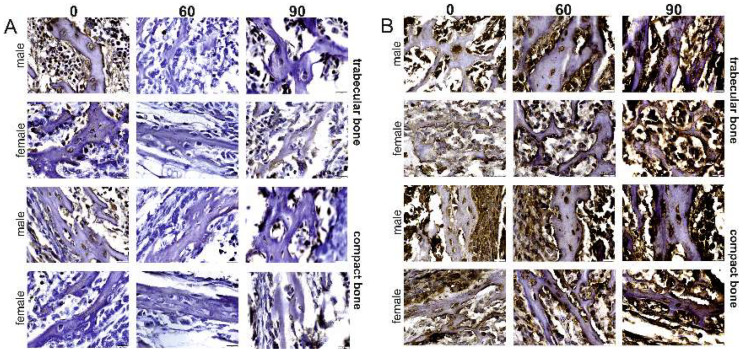
Representative pictures of the immunohistochemical analysis for osteoprotegerin (OPG; (**A**)) and receptor activator of nuclear factor kappa-Β ligand (RANKL; (**B**)) carried out on formaldehyde-fixed sections from the femoral trabecular and compact bone of male and female newborn rats prenatally exposed to 0, 60. or 90 mg/kg b.w. of fumonisins. Scale bars are equal to 20 µm. M, male; F, female. Sample pictures of the trabecular and compact bone with marked periosteum, bone marrow cavity, and trabecula are presented in the Supplementary Material, Figure S2.

A visible, positive cytoplasmic reaction for OPG in trabecular bone was observed in all the osteocytes, as well as in the matrix of female rats in the control group (Figure 4A). Many of the bone marrow cells also displayed a positive OPG immunostaining reaction. Although the OPG immunoreaction was present in numerous osteocytes and bone marrow cells in females that received the 60 mg/kg FBs’ dose, the intensity of these reactions was very weak. A similar, weak OPG immunostaining reaction was also observed in the trabecular matrix of this group. A cytoplasmic OPG immunostaining reaction was slightly visible in very few of the osteocytes in the females that received the 90 mg/kg b.w. FBs’ dose. The same weak-intensity OPG reaction was also observed in the bone marrow cells and trabecular matrix of this group.

With regards to the male rats, the cytoplasmic OPG reaction was very visible and of a strong intensity in all the osteocytes and bone marrow cells of rats in the control group, while the intensity of this reaction in the matrix varied from weak to strong (Figure 4A). The intensity of the OPG immunoreaction in males from the 60 mg/kg b.w. FBs’ group was very weak and not very visible in most of the osteocytes and bone marrow cells. Moreover, the intensity of the OPG immunostaining reaction was also very weak in the matrix. The OPG reaction was visible in both the osteocytes and bone marrow cells of the rats in the 90 mg/kg b.w. FBs’ group. The reaction was very weak in the matrix, with a very visible, intense, small line of the reaction observed in the periterritorial area of the osteocytes.

The control rats displayed a very weak RANKL immunostaining reaction in all osteocytes and bone marrow cells, as well as within the matrix, while the RANKL-positive reaction was of a higher intensity along the edge of the trabeculae. Females intoxicated with FBs displayed a very strong cytoplasmic reaction in both the osteocytes and bone marrow cells, as well as in the matrix (Figure 4B). Male rats from the control group displayed a strong RANKL immunostaining reaction in the osteocytes and bone marrow cells, with a weaker reaction observed in the matrix and along the edge of the trabeculae. The RANKL immunoreaction in males from the 60 mg/kg b.w. FBs’ group was of varied intensity, with it being diffuse in the matrix and very strong along the edge of the trabeculae, in numerous bone marrow cells. The RANKL immunostaining reaction in rats from the 90 mg/kg b.w. FBs’ group was very visible and strong in the cells, the matrix, and along the edge of the trabeculae.

In the case of the compact bone, the intensity of the OPG immunostaining reaction in female, control rats ranged from weak to a very strong reaction in numerous osteocytes, as well as a very visible reaction in the matrix. Female rats that received the 60 mg/kg b.w. FBs’ dose displayed a weak OPG reaction overall, while the reaction varied from very weak to somewhat more visible in the osteocytes, and weak in the matrix of the female rats in the 90 mg/kg b.w. FBs’ group. In the male, control rats, the intensity of the OPG reaction ranged from weak to very visible in the osteocytes, with a very weak OPG staining intensity in the matrix. Males that received the 60 mg/kg b.w. FBs’ dose displayed a very visible OPG reaction, of similar intensity, throughout the cytoplasm of all osteocytes and a weak but visible reaction in the matrix of the compact bone. A weak but visible OPG reaction was observed in numerous osteocytes of the male rats from the 90 mg/kg b.w. FBs’ group, with a varied reaction intensity in the matrix.

Females in the control group displayed a very visible RANKL reaction with varied intensity in all osteocytes and in the matrix of the compact bone (Figure 4B). The RANKL immunoreaction ranged from weak to very visible in all osteocytes and weak to not very visible in the matrix of the compact bone from females in the 60 mg/kg b.w. FBs’ group, while those in the 90 mg/kg b.w. FBs’ group displayed a strong RANKL reaction in both the osteocytes and the matrix. A very visible cytoplasmic reaction was observed in all the osteocytes of male rats in the control group, with a weak reaction observed in the matrix. The RANKL immunoreaction ranged from weak but very visible to very strong in all osteocytes and very visible in the matrix of males in the 60 mg/kg b.w. FBs’ group, while a very strong RANKL reaction was observed in all osteocytes and was very visible in the matrix of males in the 90-mg.kg b.w. FBs’ group.

### 2.3. Western-Blot Protein Expression

**Figure 5:**
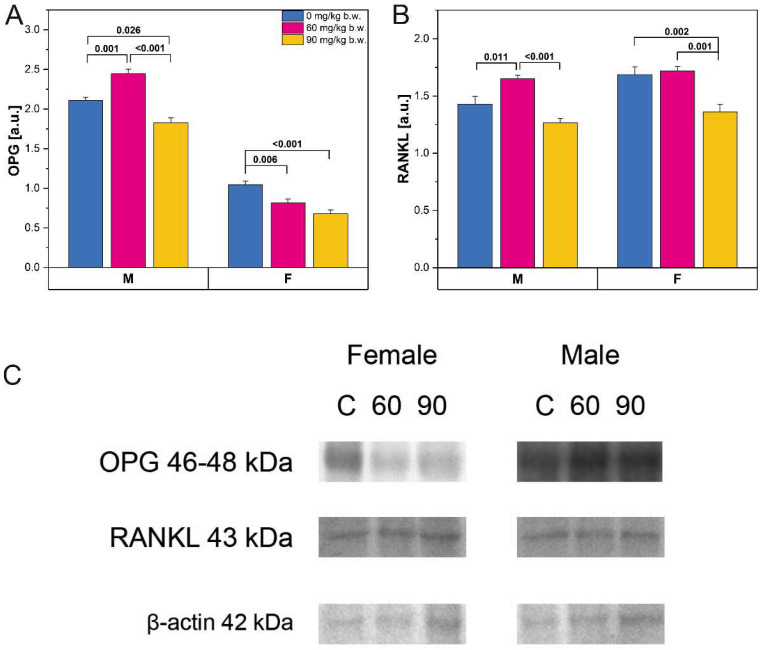
The Western-blot protein expression of (**A**): osteoprotegerin (OPG) and (**B**): RANKL from the whole bone of male and female newborn rats prenatally exposed to fumonisins. The relative abundance of OPG and RANKL was evaluated densitometrically and expressed as the ratio relative to β-actin; (**C**): representative Western blots of examined proteins in femora obtained from male and female newborn rats prenatally exposed to 0, 60, or 90 mg/kg b.w. FBs. Data are presented as mean ± standard error. M, male; F, female. Original Western blot membranes are presented in Supplementary Material, Figure S1.

Western-blot protein expression for osteoprotegerin (OPG) and receptor activator of nuclear factor-kappa-Β ligand (RANKL) (Figure 5A,B respectively) was significantly increased in male rats following consumption of the 60 mg/kg b.w. FBs’ dose, whereas the 90 mg/kg b.w. FBs’ dose caused a decrease in OPG and RANKL protein expression. OPG expression was decreased in female rats following FBs intoxication, regardless of dose, while RANKL protein expression was significantly decreased in the female rats in the 90 mg/kg b.w. group, compared to those in the control group and in the 60 mg/kg b.w. FBs’ group.

**Figure 6:**
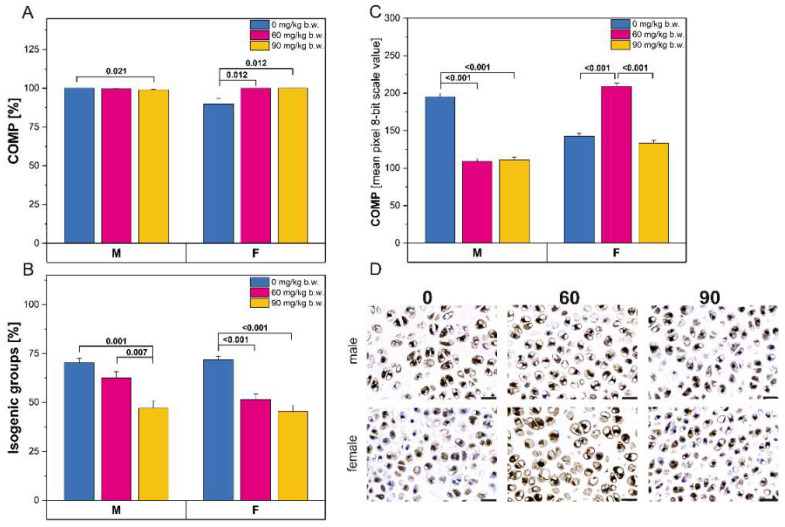
(**A**)The percentage of cells with a positive response for cartilage oligomeric matrix protein (COMP); (**B**) the percentage of isogenic groups in articular cartilage; (**C**) the intensity of the immunoreaction for COMP in articular cartilage; (**D**) representative pictures of the immunohistochemical analysis of COMP carried out on formaldehyde-fixed sections from the femoral articular cartilage of male and female newborn rats prenatally exposed to fumonisins. Scale bars are equal to 20 µm. M, male; F, female.

Following FBs intoxication, both the percentage of cells with a positive reaction (Figure 6A) and the intensity of the immunostaining reaction for cartilage oligomeric matrix protein (COMP; Figure 6C) were significantly decreased in male rats. Female rats displayed a significantly increased percentage of COMP-positive cells following consumption of FBs, regardless of dose. The intensity of the COMP immunostaining was only significantly increased compared to control rats in the rats in the 60 mg/kg b.w. FBs’ group. Interestingly, a decrease in the number of isogenic groups was observed following FBs intoxication for both sexes (Figure 6B).

## 3. Discussion

FBs inhibit ceramide synthases, which are essential enzymes in the metabolism of sphingolipids and affect many signaling systems involved in cellular growth and differentiation. Inhibition of these enzymes leads to a reduction in weight gain and feed efficiency and damage to the enteric nervous system, as well as harmful effects with regards to the morphology and cell proliferation of the intestinal epithelium [10,26,27]. Prenatal FBs’ exposure triggers dose-dependent neural tube defects as a result of the interaction between various mycotoxin, genetic, epigenetic, and metabolic factors [28]. Animal studies that made use of a wide range (10–150 mg/kg b.w. FBs) of FBs’ doses have shown that FBs’ action is dose dependent [29,30,31,32]. As previously mentioned, FBs intoxication is associated with the stunting of growth in children and with folic acid deficiency-linked birth defects [16,17,18]. There are no data or clinical observations, however, that relate to prenatal FBs’ exposure and the effects of the exposure on the bone metabolism of newborn offspring. Animal studies on postnatal FBs’ exposure observed endosteal resorption, thinning of the bone wall, and weakening of the whole bone [33,34]. An important difference between the current study and previous animal studies involving FBs’ exposure and neural tube defects is the timing and duration of the subclinical FBs intoxication of the adult pregnant dams.

Throughout life, two main metabolic processes take place in the bones: modeling and remodeling. In childhood and adolescence, the dominant process is bone modeling, as a result of which the appropriate weight, size, and shape of the skeleton are achieved. Thus, the bone modeling process concerns bone growth in length and width. In a mature organism, the modeling processes are replaced by bone remodeling processes, including bone formation and resorption, which are designed to maintain the appropriate mass of the skeleton and also to participate in the repair of micro damages and the maintenance of calcium-phosphate homeostasis within the body [35,36,37,38]. In young, healthy bone, a fine balance is maintained between bone formation and resorption, which guarantees a relatively constant bone mass and allows for the achievement of peak bone mass, which determines physiological processes involved in age-related bone loss. Then, the bone resorption process is predominant and depends not only on genetic factors but also on nutrition, which influences all processes involved in the maintenance of bone homeostasis, on cellular and molecular levels.

Bone turnover processes are regulated by complex physiological mechanisms. Bone proteins, including osteoprotegerin (OPG), receptor activator of the nuclear factor NF-κB (RANK), and the RANK ligand (receptor activator of nuclear factor NF-κB ligand-RANKL), together play an important role in overall bone metabolism [39]. OPG is released from many cells such as osteoblasts, dendritic cells, and lymphocytes, while RANKL is detected on osteoblasts, osteoclasts, and primary mesenchymal cells surrounding cartilage and chondrocytes, as well as on endothelial cells, and in the extracellular space. RANKL plays a vital role in the differentiation of osteoclasts from precursors. It intensifies the activity and viability of these cells through the inhibition of apoptosis, thereby promoting bone resorption. This system plays a fundamental role in bone loss and osteoporosis. The development and function of osteoclasts is primarily stimulated by the interaction of RANKL with the RANK receptor on the surface of these cells. These processes are inhibited when RANKL remains bound by OPG, which also has a high affinity for the RANK receptor and is in competition with RANK [40]. The underlying cause of bone diseases of various origins is usually an imbalance between RANKL and OPG [41]. Furthermore, the correct bone metabolic processes are crucial for the proper macro- and microstructure of bones and, consequently, for adequate mechanical strength. The mechanical resistance of bone is dependent on its mineral density and mechanical properties such as stiffness or strength [42,43]. These properties are in turn influenced by genetic, environmental, geometric, physiological, and pathological factors, as well as the microarchitecture of bone trabeculae, appropriate mineralization, and the presence of micro damages.

The present study involved the use of one strain of rats that were housed under the same environmental conditions, fed the same basal diet, and, through their mothers, were exposed to different doses of FBs: 0, 60, or 90 mg/kg b.w. This maternal FBs’ exposure resulted in the inhibition of prenatal growth in the newborn offspring, in a dose- and sex-dependent manner. All newborn offspring, irrespective of FBs’ dose and rat sex, had shortened femur lengths and only the body weight of males delivered by mothers from the 60 mg/kg b.w. FBs’ group was not different from the body weight of control rats. There were also changes observed in the femoral geometry of the offspring, which were dependent on FBs’ dose. Both male and female newborn offspring from the 90 mg/kg b.w. FBs’ group had a significantly reduced cross-sectional area of the femoral midshaft. Although no significant changes in femoral cross-sectional area were observed in the 60 mg/kg b.w. FBs’ group, femora from newborns prenatally exposed to either the 60- or the 90 mg/kg b.w. FBs’ doses were weaker compared to those from the control group, as was shown by the mechanical testing. Reduced stiffness was observed in female rats, irrespective of the FB dose.

There is a dearth of information regarding the effects of prenatal mycotoxin exposure on general fetus development, including offspring bone metabolism, development, and subsequent changes in bone mechanical endurance or geometry. A previous study showed that FB1 given at a dose of 60 mg/kg b.w, between the 8th and 12th day of pregnancy, reduced relative litter weight and ossification of sternebrae and vertebrae in the offspring [44]. A study on hamsters showed that 18 mg of FB1 in water/kg b.w./day did not induce any clinical signs of FB intoxication in the mothers but resulted in reduced fetal body weight and inhibited bone ossification in the offspring [45]. Other available data relate to postnatal life of different animal models. Both aflatoxin and ochratoxin have been shown to decrease bone strength [46]. Mink dams exposed to deoxynivalenol displayed disturbances in overall bone homeostasis, with decreased mechanical endurance and reduced geometric properties of long bones, while results from other studies performed on rats and laying hens exposed to FBs were inconclusive [10,13,34]. Studies on rats or hens have also shown that FBs’ exposure can destroy the intestinal barrier [8,13,27].

Mycotoxin-induced disturbances of the intestinal barrier, as well as the intestinal epithelial morphology and microflora, can possibly cause the development of a slight gastroenteritis, which results in malnutrition and changes in blood basal morphology, as well as altered bone endurance, which was observed in our newborn rats.

Long bones undergo geometric development by increasing the cross-sectional area of their midshaft and the bone marrow cavity. The thickness of the bone wall is dependent on the formation of new layers on the periosteal side, through a process called apposition. The bone remodeling process also occurs on the endosteal side. Bone remodeling on the side of the bone marrow cavity and the process of apposition both influence the cross-sectional area of the midshaft in the long bone. If bone synthesis is inhibited or bone resorption is greater than bone synthesis, endosteal resorption is observed as an enlargement of the marrow cavity, with accompanying thinning of the bone wall and a reduced cross-sectional area [34].

The reduced bone strength observed in the rats in the current study could be a result of an impairment in collagen synthesis and other organic components of bone tissue in the fetuses as a result of the malnutrition in the mothers exposed to FBs. Structural information obtained from the analysis of collagen fibers (as a component of the bone organic phase) of newborns’ bone tissue showed that both mature and immature collagen fibers were present, with a predominance of the mature, thick collagen in compact and trabecular bone in all groups. The changes in collagen content observed in the rats were sex and dose dependent, with a very visible increase in immature and thin collagen observed in the compact bone of the male newborns. This could suggest that there was intense bone turnover that took place, including that of the matrix, which in turn could lead to disturbances in bone development and function.

Collagen fibers are necessary for hydroxyapatite deposition and bone calcification [47,48]. The reduction in the mature, thick collagen fibers resulted in decreased bone endurance and a much higher than normal content of immature collagen fibers, which in turn can result in the development of bone deformities and locomotive dysfunctions [49].

Mycotoxins are causative factors in the imbalance and disturbance of vitamin D metabolism, which adversely affects leg bone development in broiler chickens, triggering tibial dyschondroplasia [50]. A study performed on growing broiler chickens proved that FBs exert a toxic effect on chondrocytes isolated from the epiphyseal growth plate [51].

As mentioned above, the RANK/RANKL/OPG system is very important for bone formation and the maintenance of bone mass. The current study showed that the FBs-induced changes in expression of OPG and RANKL were dose and sex dependent. Analysis of the OPG immunolocalization showed that the intensity of its reaction was also dependent on the part of the bone that was analyzed. The 60 mg/kg b.w. FBs’ dose did not significantly affect the activity of proliferating chondrocytes in the growth plate of male rats, as well as the activity of chondrocytes in the articular cartilage. In the female newborns, chondrocyte activity was decreased following FBs intoxication (both doses), as indicated by the weaker OPG immunoreaction. Moreover, the number of OPG immunoreactive chondrocytes decreased in both parts of the bone in females, although it was dependent on the area of detection in both cartilages. The increase in OPG expression observed could be a positive effect, considering the fact that OPG is central to the regulation of bone-related disease, but the RANKL/OPG ratio is of most importance. Our study indicated that RANKL expression also increased in male newborns following consumption of the 60 mg/kg b.w. FBs’ dose. On the other hand, in the female newborns, OPG expression decreased, while RANKL expression was significantly increased compared to the OPG expression.

An increase in OPG concentration is observed with age and in women with increased bone turnover. In general, it is a compensatory response to increased resorptive activity [52]. Increased concentrations of RANKL have been observed in the bone marrow cells (a potential source of osteoblast precursors) and lymphocytes of postmenopausal women [53]. OPG has been shown to be expressed in the stromal cells, lymphocytes, and epithelial cells, except for the osteoblasts and osteocytes, while RANK is expressed in osteoclast precursors, mature osteoclasts, dendritic cells, macrophages, microglia, osteoblasts, and to a lesser extent in osteocytes [54]. In the case of bone pathology, the balance between RANKL and OPG, as essential factors that determine the number and activity of osteoclasts, is important and serves as a useful biomarker. This balance reflects both bone formation and bone resorption processes. The intensity and immunolocalization of the OPG and RANKL reactions in the rats in the current study showed that this balance does not always occur in all areas of the bone, although the overall expression (determined by WB) could be considered as in balance.

Chondrocytes are important in synthesizing and maintaining components of the extracellular matrix. They are also present in articular cartilage. At the periphery, young chondrocytes (called chondroblasts) have an elliptical shape and are placed with their long axis parallel to the surface, while mature chondrocytes have a rounded shape and often appear as an isogenous group that originates from the mitotic divisions of a single chondrocyte [55]. Although articular cartilage is considered to be a structure with low regenerative capacity (complete regeneration is rarely observed, with inter-patient variability), some metabolic and enzymatic activity exists and chondrocytes are able to proliferate and synthesize extracellular matrix; however, the process is dependent on having enough new chondrocytes and their migration [56]. The number and shape of the isogenous groups reflect the response of the chondrocytes to age-related changes and injury [57].

The current study showed that the percentage of isogenous groups in the middle (or transitional) zone, per vertical section of non-calcified and non-weight-bearing femoral cartilage, declined with FBs’ dose irrespective of the sex of the rats, indicating the probable harmful effects of the FBs on the cartilage, observed as the inhibition of the proliferation of the chondrocytes.

Furthermore, cartilage oligomeric matrix protein (COMP), which is found primarily in cartilage and is responsible for binding calcium and the interaction with other matrix proteins, has been shown to stimulate collagen fibril formation in growing subjects [58]. COMP is significantly upregulated in the early stages of osteoarthritis, in an apparent attempt to repair the cartilage; and, since it is a cartilage turnover marker, it also increases concomitantly in people with early stages of osteoarthritis [59,60]. The articular cartilage plays a key role in the functioning of the joints, including the uptake and distribution of the load.

Our study showed that the male newborn rats were unaffected by the mycotoxins, irrespective of the FBs’ dose, as the intensity of the COMP immunoreaction was significantly weaker compared to that observed in the control group, while the percentage of COMP-positive chondrocytes increased with FBs’ dose in the female newborns, with the intensity of the COMP immunoreaction being highest in the 60 mg/kg b.w. FBs’ group, indicating enhanced matrix turnover and an apparent attempt at repair (as mentioned above).

## 4. Materials and Methods

The experiment was performed in accordance with EU Directive 2010/63/EU under the license of the State Scientific Research Control Institute of Veterinary Medicinal Products and Feed Additives in Lviv, Ukraine.

### 4.1. Animals and Experimental Design

Eighteen pregnant (5 weeks old) Wistar rat dams were housed individually in polypropylene cages (with the dimensions of 380 × 200 × 590 mm) and allowed a 1-week acclimatization period, during which they were accustomed to the laboratory conditions. The dams were kept at a temperature of 21 ± 3 °C, humidity of 55 ± 5%, with a 12-h/12-h day/night cycle and had free access to water. After the acclimatization period, the rats were randomly allocated to either a control group (the 0 FB group; *n* = 6), not treated with FBs, or to one of the two groups intoxicated with FBs, either at 60 mg/kg b.w. FBs (60 FB group) or at 90 mg/kg b.w. FBs (90 FB group); each group consisted of six dams. The pregnant dams were fed a standard laboratory rodents’ diet ad libitum. Fumonisins were administered daily, intragastrically (75% FB1 and 25% FB2, respectively). The FBs were administered in 0.5 mL of 0.9% saline solution from the seventh day of pregnancy to parturition, by oral gavage, as previously described [10]. The FBs’ preparation has also been described previously [13]. Control rats received saline solution in the corresponding amount and via the same route of administration. All pregnant dams had similar body weight at the beginning of the experiment. During the experiment, the body weight gain was monitored, and all dams increased in weight in a similar manner. For the first 8 days of pregnancy, their body weight gain was approximately about 2–4 g per day; during the next days, it was in the range of 8–9 g and always was comparable between groups. We did not observe a change in water and food intake. No alterations in behavior or in dams’ basal health under veterinarian examination were observed.

The 90 mg/kg b.w. FBs’ dose was equal to 0.1 of the established LD50 value, which is sufficient to induce subclinical intoxication when given to adolescent rats for 21 days [10,27]. The 60 mg/kg b.w. FBs’ dose was higher than that required to trigger embryonic neural tube defects, when given during early pregnancy, and was equal to 1/15 of the established LD50 value (these doses did not induce even subclinical intoxication when they were given to adolescent rats for 21 days) [10,32]. On the day of parturition, newborns from each group (*n* = 6 males; *n* = 6 females) were weighed and euthanized by CO_2_ inhalation, and they were subjected to further analysis.

After the birth, randomly selected male and female newborns, belonging to the same group as their mother, were included in the study. After euthanasia, both femora were dissected from the newborn rats and the remaining soft tissues were removed using a scalpel blade. After measuring the length of the right femora, mechanical testing was performed and then the bones were fixed in phosphate-buffered 4% (*v*/*v*) paraformaldehyde (pH 7.0) and subjected to immunohistochemical analysis. The left femora were immediately frozen in liquid nitrogen and stored at −80 °C until they were subjected to Western blot analysis.

### 4.2. Mechanical Testing

Right femora were subjected to the three-point bending test on a universal testing machine (Zwick-Roell 010, Zwick-Roell GmbH & Co., Ulm, Germany). Bones, placed on two support points, positioned apart at 40% of the total bone length, were loaded in bending at the midpoint of the bone mid-shaft with a constant load rate of 2 mm/min until fracture. On the basis of recorded load-deformation curves, the following bone mechanical properties were determined: the ultimate force, as the force causing bone fracture; stiffness was measured as the slope of the elastic part of the load-displacement curve; bending moment was calculated using an appropriate equation. On the basis of bone mid-diaphysis geometry, the cross-sectional area of the mid-shaft was determined [10,61].

### 4.3. Immunohistochemistry

Immunohistochemical staining was performed according to the manufacturer’s protocols (Abcam, Cambridge, UK). For immunohistochemistry, rabbit polyclonal to osteoprotegerin (OPG; ab73400) and cartilage oligomeric matrix protein (COMP, E-AB-14886, Biorbyt, Wuhan, China) antibodies and mouse monoclonal to receptor activator for nuclear factor kappa-Β ligand (RANKL; ab239607) antibodies, diluted in Diamond antibody diluent (Cell Marque Corp., Rocklin, CA, USA), were used as primary antibodies. Ready-to-use Bright Vision + Poly-HRP-Anti Ms/Rb IgG Biotin-free (Immunologic, Duiven, The Netherlands) served as the secondary antibody. The 3,3′-diaminobenzidine tetrahydrochloride (DAB, D5905, Sigma-Aldrich, St. Louis, MO, USA) was used as a substrate-staining chromogen; counterstaining was performed with Mayer’s hematoxylin (MHS32-1L, Sigma-Aldrich, St. Louis, MO, USA) [62,63].

The intensity of immunoreaction was measured both by determining the percentage of cells with a positive response and by the quantitative comparison of mean pixel intensity in the photomicrographs, which were first converted into negatives and then into 8-bit grayscale digital images, with a scale from 0 (white pixel) to 255 (black pixel), where the higher the pixel value, the higher the intensity of the immunohistochemical reaction [25,63]. The intensity of the immunoreaction in each of the analyzed images was measured in six randomly selected areas of the positive signal. The analyses were done blindly using ImageJ software (ver. 1.53c, Rasband, W.S., National Institutes of Health, Bethesda, MD, USA) [64].

The number of isogenous groups of chondrocytes and single located chondrocytes was calculated in articular cartilage and expressed as a percentage of isogenous groups. A cluster was considered as an isogenous group (all formed through division of a single progenitor cell of chondrocytes).

### 4.4. Western Blot

The left femora that were stored at −80 °C were removed from the freezer and placed into a mortar and pestle kept on dry ice. The bones were then homogenized in lysis buffer (125 mM TRIS-HCl pH 6.8; 4% SDS; 10% glycerol; 100 mM DTT), boiled in a water bath for 10 min, and centrifuged at 13,000× *g* for 15 min. The supernatant was then removed into new Eppendorf tubes and the pellet was discarded. The Bradford method [65] was used to determine the protein content. Samples containing 80 µg of protein were separated by 12% SDS-PAGE and then electroblotted onto an Immobilon P membrane (Sigma-Aldrich, St. Louis, MO, USA). After the transfer, the membranes were blocked with 3% low-fat milk in PBS for 1 h and incubated overnight with the primary antibodies. The same OPG and RANKL antibodies as those used for the immunohistochemistry were also used for the Western blots. The membranes were washed three times for 10 min with PBS containing 0.05% TRITON X-100 (Sigma-Aldrich, St. Louis, MO, USA) and incubated for 2 h in the presence of a 1:30,000 dilution of alkaline phosphatase-conjugated goat anti-rabbit IgG (Abcam, Cambridge, UK). The membranes were visualized after addition of BCIP (5-bromo-4-chloro-3indolyl phosphate, Sigma-Aldrich, St. Louis, MO, USA) and NBT (nitrotetrazolium blue chloride, Sigma-Aldrich, St. Louis, MO, USA), which gives a blue reaction color. An anti-β-actin antibody (1:2000, Sigma-Aldrich, St. Louis, MO, USA) was used as the loading control. The blots were densitometrically quantified and normalized to their corresponding β-actin bands. The quantitative analysis of protein bands was performed using the public domain ImageJ program with “Gel Analysis” [64]. Three independent experiments were performed.

### 4.5. Statistical Analysis

The statistical analysis was performed using Statistica 13.1 (Tibco Software, Palo Alto, CA, USA) and Origin 2021b (OriginLab, Northampton, MA, USA). Normality was assessed by the Shapiro–Wilk test, while the homogeneity of the variance was studied using the Levene test. A one-way ANOVA with the Fumonisin treatment as the main effect was used to assess the changes under the influence of Fumonisin administration. Non-parametric data were analyzed using a Kruskal–Wallis H test. Post hoc tests (Tukey or Dunn’s) were applied to evaluate differences in the analyzed parameters between the control and supplemented groups. For all tests, a *p*-value < 0.05 was established as statistically significant. All the data are reported as mean ± standard error. Analysis was performed separately for each sex.

## 5. Conclusions

The immunolocalization of OPG and RANKL performed in bone and articular cartilage indicated that the effects observed resulted from the FBs-induced alterations in bone metabolism. It was also proven by the changes in WB expression of OPG and RANKL. It can be concluded that the negative effects of prenatal FBs’ exposure on general growth and bone morphometry are a result of the altered expression of proteins responsible for bone metabolism and were dose and sex dependent. Of course, used doses cannot be directly extrapolated to humans, and other preclinical studies, using different animal models, are required.

## Figures and Tables

**Table 1:** Body weight, femur geometry, mechanical properties, and immature collagen content of fumonisin-intoxicated male and female rats. Data are presented as mean ± SE. Statistically significant differences between groups (at *p* < 0.05) are indicated by ^a^ and ^b^.

Dependent Variable	Sex	FBs [mg/kg b.w.]			*p*-Value
		0	60	90	
Body weight [g]	M	10.050 ± 0.438 ^a^	6.819 ± 0.105 ^b^	6.839 ± 0.203 ^b^	<0.001
Femur length [mm]		11.546 ± 0.068 ^a^	10.794 ± 0.245 ^b^	10.200 ± 0.214 ^b^	<0.001
Cross-sectional area [mm^2^]		4.148 ± 0.194 ^a^	3.935 ± 0.288 ^a,b^	3.250 ± 0.212 ^b^	0.034
Ultimate force [N]		1.693 ± 0.058 ^a^	1.190 ± 0.096 ^b^	1.170 ± 0.028 ^b^	<0.001
Stiffness [N/mm]		5.590 ± 0.409 ^a^	3.050 ± 0.163 ^b^	3.060 ± 0.127 ^b^	<0.001
Collagen_trabecular_ [%]		13.188 ± 1.782 ^a,b^	15.948 ± 1.867 ^a^	8.003 ± 0.434 ^b^	0.004
Collagen_compact_ [%]		20.051 ± 2.034 ^a^	7.589 ± 0.978 ^b^	17.365 ± 1.528 ^a^	<0.001
Body weight [g]	F	7.766 ± 0.202 ^a^	6.747 ± 0.215 ^a,b^	5.903 ± 0.427 ^b^	0.001
Femur length [mm]		10.929 ± 0.075 ^a^	9.870 ± 0.174 ^b^	9.769 ± 0.173 ^b^	<0.001
Cross-sectional area [mm^2^]		3.945 ± 0.219 ^a^	3.726 ± 0.283 ^a,b^	3.115 ± 0.142 ^b^	0.040
Ultimate force [N]		1.434 ± 0.099 ^a^	1.104 ± 0.101 ^b^	1.212 ± 0.082 ^b^	0.012
Stiffness [N/mm]		4.508 ± 0.743	2.636 ± 0.324	3.770 ± 0.405	0.065
Collagen_trabecular_ [%]		14.231 ± 1.144 ^a,b^	13.634 ± 0.661 ^a^	18.699 ± 1.967 ^b^	0.032
Collagen_compact_ [%]		8.283 ± 0.764 ^a^	17.041 ± 3.056 ^b^	18.165 ± 1.782 ^b^	0.006

FBs, Fumonisins; SE, standard error; F, female; M, male.

## Data Availability

The data presented in this study are available on request from the corresponding author.

## Supplementary Materials

The following are available online at https://www.mdpi.com/article/10.3390/ijms222413234/s1.
