# Supplementary material for: The Influence of Prenatal Fumonisin Exposure on Bone Properties, as well as OPG and RANKL Expression and Immunolocalization, in Newborn Offspring Is Sex and Dose Dependent
Source: Int J Mol Sci. 2021 Dec 8;22(24):13234. doi: 10.3390/ijms222413234 (PMC8705866; doi:10.3390/ijms222413234)
Supplement: Supplementary file 1 [file ijms-22-13234-s001.zip › ijms-1469235-Supplementary Figure S1.pdf]

SUPPLEMENTARY MATERIAL

The Influence of Prenatal Fumonisins Exposure on Bone Properties, OPG, RANKL Expression and Immunolocalization in Newborn Offspring Is Sex- and Dose-Dependent

Ewa Tomaszewska [1,*], Halyna Rudyk [2], Izabela Świetlicka [3,*], Monika Hułas-Stasiak [4], Janine Donaldson [5], Marta Arczewska [3], Siemowit Muszyński [3], Piotr Dobrowolski [4], Iwona Puzio [1], Volodymyr Kushnir [2], Oksana Brezvyn [2], Viktor Muzyka [2] and Ihor Kotsyumbas [2]

[1] Department of Animal Physiology, Faculty of Veterinary Medicine, University of Life Sciences in Lublin, Akademicka St. 12, 20-950 Lublin, Poland; ewaRST@interia.pl (E.T.); iwona.puzio@up.lublin.pl (I.P.)
[2] State Scientific Research Control Institute of Veterinary Medicinal Products and Feed Additives, Donetska St. 11, 79000 Lviv, Ukraine; galusik.77@gmail.com (H.R.); wolodjak@gmail.com (V.K.); brezvun@gmail.com (O.B.); muzyka@scivp.lviv.ua (V.M.); dir@scivp.lviv.ua (I.K.)
[3] Department of Biophysics, Faculty of Environmental Biology, University of Life Sciences in Lublin, Akademicka St. 13, 20-950 Lublin, Poland; izabela.swietlicka@up.lublin.pl (I.Ś.); marta.arczewska@up.lublin.pl (M.A.); siemowit.muszynski@up.lublin.pl (S.M.)
[4] Department of Functional Anatomy and Cytobiology, Faculty of Biology and Biotechnology, Maria Curie-Sklodowska University, 19 Akademicka St., 20-033 Lublin, Poland; piotr.dobrowolski@umcs.lublin.pl (P.D.)
[5] School of Physiology, Faculty of Health Sciences, University of the Witwatersrand, 7 York Road, Parktown, Johannesburg, 2193, South Africa; janine.donaldson@wits.ac.za (J.D.)
* Correspondence: ewaRST@interia.pl (E.T.); izbela.swietlicka@up.lublin.pl (I.Ś.)

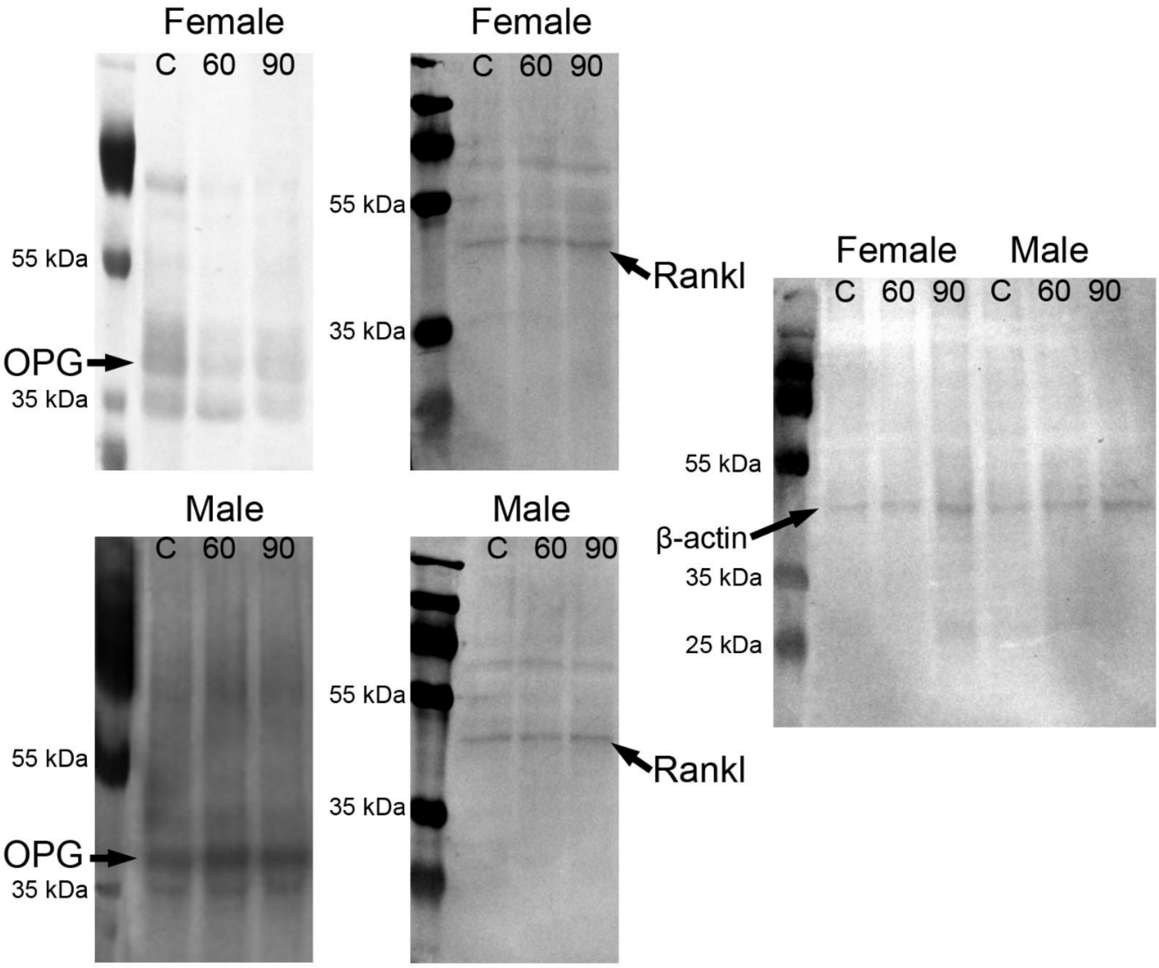

Figure S1. Representative, original Western blot membranes presenting the level osteoprotegerin (OPG) and receptor activator of nuclear factor-kappa-B ligand (RANKL) as well as β-actin as representative loading control.
